# Structure-Based Discovery of MolPort-137: A Novel Autotaxin Inhibitor That Improves Paclitaxel Efficacy

**DOI:** 10.3390/ijms26020597

**Published:** 2025-01-12

**Authors:** Prateek Rai, Christopher J. Clark, Vandana Kardam, Carl B. Womack, Joshua Thammathong, Derek D. Norman, Gábor J. Tigyi, Kevin Bicker, April M. Weissmiller, Kshatresh Dutta Dubey, Souvik Banerjee

**Affiliations:** 1 Molecular Biosciences, Middle Tennessee State University, Murfreesboro, TN 37132, USA; pr3x@mtmail.mtsu.edu (P.R.); cjc8g@mtmail.mtsu.edu (C.J.C.); kevin.bicker@mtsu.edu (K.B.); april.weissmiller@mtsu.edu (A.M.W.); 2 Department of Chemistry, Middle Tennessee State University, Murfreesboro, TN 37132, USA; jt8p@mtmail.mtsu.edu; 3 Department of Chemistry, Shiv Nadar Institution of Eminence, Delhi 201314, India; vk935@snu.edu.in; 4 Department of Biology, Middle Tennessee State University, Murfreesboro, TN 37132, USA; cbw4@mtmail.mtsu.edu; 5 Department of Physiology, University of Tennessee Health Science Center, Memphis, TN 37132, USA; dnorman7@uthsc.edu (D.D.N.); gtigyi@uthsc.edu (G.J.T.)

**Keywords:** autotaxin, LPA signaling, virtual screening, molecular simulations, cancer therapy resistance, combination therapy

## Abstract

The autotaxin–lysophosphatidic acid receptor (ATX-LPAR) signaling axis is pivotal in various clinical conditions, including cancer and autoimmune disorders. This axis promotes tumorigenicity by interacting with the tumor microenvironment, facilitating metastasis, and conceding antitumor immunity, thereby fostering resistance to conventional cancer therapies. Recent studies highlight the promise of ATX/LPAR inhibitors in combination with conventional chemotherapeutic drugs to overcome some forms of this resistance, representing a novel therapeutic strategy. In the current study, we employed structure-based virtual screening, integrating pharmacophore modeling and molecular docking, to identify MolPort-137 as a novel ATX inhibitor with an IC_50_ value of 1.6 ± 0.2 μM in an autotaxin enzyme inhibition assay. Molecular dynamics simulations and binding free energy calculations elucidated the binding mode of MolPort-137 and its critical amino acid interactions. Remarkably, MolPort-137 exhibited no cytotoxicity as a single agent but enhanced the effectiveness of paclitaxel in 4T1 murine breast carcinoma cells and resensitized taxol-resistant cells to paclitaxel treatment, which highlights its potential in combination therapy.

## 1. Introduction

**Figure 1:**
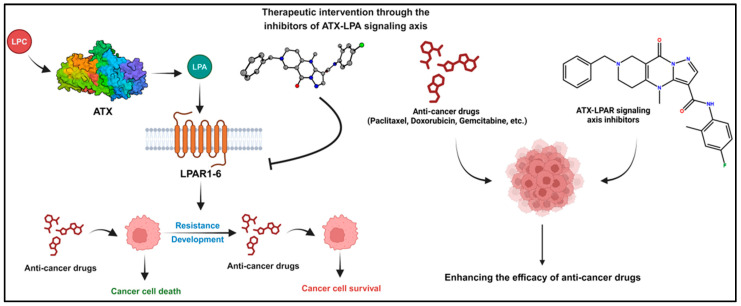
Employing inhibitors affecting the ATX-LPAR pathway to overcome resistance in cancer therapy.

Autotaxin (ATX) is a secreted lysophospholipase D responsible for producing the extracellular bioactive lipid mediator lysophosphatidic acid (LPA) through the hydrolysis of lysophosphatidylcholine (LPC). As a member of the ectonucleotide pyrophosphatase/phosphodiesterase 2 (ENPP2) family, ATX plays a pivotal role in various cellular responses, including cell proliferation, motility, survival, and migration, by modulating specific G protein-coupled lysophosphatidic acid receptors (LPAR1-6) [1,2]. The ATX-LPAR signaling pathway has been associated with the pathogenesis of numerous clinical conditions, including cancer, neuropathic pain, autoimmune disorders, and cardiovascular diseases [3,4]. Aberrant ATX expression has been observed in several tumor types, such as hepatocellular carcinoma, renal cell carcinoma, melanoma, lung cancer, and glioblastoma [5,6,7,8,9,10]. Although breast cancer cells do not exhibit abnormal ATX expression relative to normal breast tissue, the ATX-LPAR signaling axis significantly enhances the tumorigenicity of breast cancer, likely due to ATX from the tumor microenvironment or stromal ATX [11,12]. Recent studies suggest that secreted ATX exerts a dual pro-tumorigenic function: inhibiting antitumor immunity by preventing cytotoxic CD8^+^ T cell infiltration [13,14] and promoting migration and metastasis via various LPARs [15,16,17]. The ATX-LPAR signaling axis has been identified as a critical player in resistance, with increased expression of ATX observed in cancer treated with radiation and chemotherapy. Consequently, targeting the ATX-LPAR signaling axis represents a central strategy for overcoming cancer therapy resistance (Figure 1). Numerous studies in the literature have demonstrated the potential of ATX-LPA signaling axis inhibitors in combination therapies to enhance cancer treatment outcomes and overcome resistance. For instance, the ATX inhibitor GLPG1690 has shown synergistic effects with doxorubicin, reducing tumor growth in the 4T1 breast cancer model [18]. Additionally, novel benzenesulfonamide analogs acting as ATX antagonists have effectively reduced paclitaxel resistance in murine breast cancer cells [19]. The combination of the ATX inhibitor ONO-8430506 with doxorubicin reduced tumor growth and metastasis by more than 70% in an in vivo 4T1 breast cancer model, showing a significant synergistic effect [20]. LPA signaling stabilizes the transcription factor NRF2, which initiates the antioxidant response and promotes proteins that protect cancer cells from oxidative damage [20]. Also, LPA-induced NRF2 expression enhances the transcription of multidrug-resistant transporters and antioxidant genes by 2- to 4-fold, thereby protecting cells from doxorubicin-induced death [21]. Furthermore, LPA signaling mitigates the pro-apoptotic effects of ceramides, which are crucial in the therapeutic actions of many chemotherapeutic drugs and in radiation therapy. By enhancing ERK and PI3K activities, LPA provides survival signals and decreases ceramide formation, reducing the potential for ceramides to block pro-survival pathways [21]. LPA also prevents cancer cells from undergoing apoptosis caused by histone deacetylase (HDAC) inhibitors by activating HDAC [22].

Recent studies have highlighted the potential benefits of simultaneously targeting both ATX and LPAR to enhance the efficacy of cancer treatments [23,24]. This dual approach disrupts the signaling cascade at multiple points, potentially overcoming some forms of drug-resistant cancer and improving therapeutic outcomes. Extensive research has been focused on developing small-molecule non-lipid ATX inhibitors. ATX contains a three-part binding site that includes an enzymatic spot containing a couple of zinc ions, an adjoining hydrophobic pocket, and a tunnel. Existing ATX inhibitors are classified into five categories: Class I inhibitors (orthosteric inhibitors), which occupy the catalytic site and hydrophobic pocket, mimicking the natural substrate LPC (e.g., PF-8380) [25], but none have advanced through clinical trials, likely due to their high partition coefficient; Class II inhibitors (non-carboxylic acid small-molecule hydrophobic pocket-only inhibitors), which competitively impede LPC binding to ATX (e.g., 3b and GRI-918013) [19,26], but they are not currently in clinical trials; Class III inhibitors (allosteric inhibitors), which bind to the tunnel and non-competitively obstruct the transport and release of LPA (e.g., PAT-347) [27]; Class IV inhibitors (pocket-tunnel hybrid inhibitors), which combine features of Class II and Class III inhibitors to competitively thwart LPC binding (e.g., GLPG-1690) [28]; and Class V inhibitors (steroid-based inhibitors) [29], which have been recently developed to occupy the tunnel and catalytic site, aiming to prevent LPC binding and/or impede LPA release without interfering with the catalytic site. The primary objective of these inhibitors is to block LPC binding and LPA release, thereby mitigating the role of ATX in cancer progression and other pathological conditions. Zhang et al. reviewed and summarized various ATX inhibitors, classifying them based on their binding modes to the ATX tripartite binding site [30]. Similarly, several LPAR1 antagonists have also been assessed for their efficacy in cancer treatment [8,31,32].

**Figure 2:**
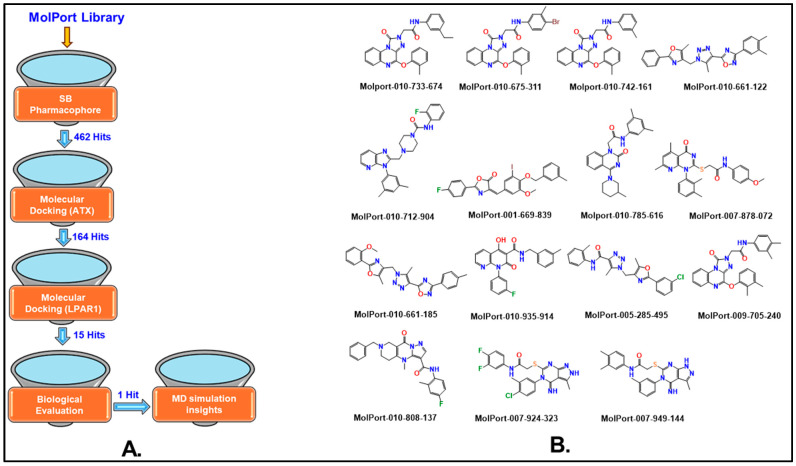
(**A**) Workflow of the virtual screening process used in this study. (**B**) The top 15 compounds were purchased and biologically evaluated for ATX enzyme inhibition. (SB stands for structure-based). Conventional colors of elements were used to represent nitrogen in blue, oxygen in red, sulfur in yellow, chlorine, and fluorine in green.

In this study, our primary objective was to delineate a computational drug discovery paradigm for identifying inhibitors targeting the ATX-LPAR (particularly LPAR1) signaling axis (Figure 2). We utilized a hierarchical virtual screening strategy that combined pharmacophore-based screening and molecular docking. The potential inhibitors identified through this process were further evaluated using an in vitro autotaxin enzyme inhibition assay. In this assay, 15 selected compounds were tested at a fixed concentration of 10 µM. This approach yielded one hit compound, MolPort-137, demonstrating ~76% inhibition at 10 µM. MolPort-137 was subsequently tested in combination with paclitaxel (PTX) using a cell viability assay on both wild-type and taxol-resistant 4T1 murine breast cancer cell lines. The findings demonstrated that PTX’s anticancer efficacy was improved in both cell lines. We also conducted molecular dynamics (MD) simulations to observe the protein–ligand interaction dynamics and the stability of MolPort-137 within its target binding site over time. We employed molecular mechanics/generalized Born surface area (MM-GBSA) calculations to quantify the binding free energy, estimating the interaction strength between MolPort-137 and its target. Additionally, computational alanine scanning mutagenesis was performed to identify key residues critical for binding by systematically mutating them to alanine and assessing the mutation’s impact on binding affinity.

## 2. Results and Discussion

### 2.1. Pharmacophore-Based Virtual Screening

**Figure 3:**
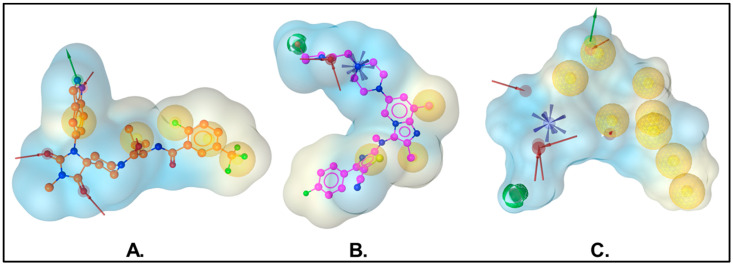
SB pharmacophore models generated using the software package LigandScout. (**A**) SB pharma-cophore model for the protein–ligand complex with PDB ID: 6W35. (**B**) SB pharmacophore model for the protein–ligand complex with PDB ID: 5MHP. (**C**) The final merged-feature pharmacophore model consisting of 17 features: eight hydrophobic features, two hydrogen bond donors, six hy-drogen bond acceptors, and one positive ionizable feature. Exclusion volumes are omitted. 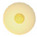: hydrophobic interaction, 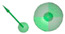: hydrogen bond donor, 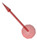: hydrogen bond acceptor, and 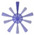: positive ionizable area.

Pharmacophore modeling is essential in the virtual screening process for identifying potential drug candidates. A pharmacophore describes the spatial arrangement of features crucial for optimal interactions with a specific biological target, which are fundamental for the biological studies of the compound. This approach effectively narrows down large chemical libraries to a manageable number of promising compounds, thereby accelerating the drug discovery process [33,34]. In this study, we followed the SB pharmacophore creation guidelines available in LigandScout to generate pharmacophores representing non-bonding interactions of two individual ATX protein–ligand complexes, 6W35 and 5MHP. The number of pharmacophoric features varied, with ten features for 6W35 and seven for 5MHP (Figure 3). We aligned the two SB pharmacophores using 6W35 as the reference point. This method allowed us to generate a shared feature pharmacophore, which was then merged to interpolate overlapping features. The final pharmacophore model comprised 17 features: eight hydrophobic features, two hydrogen bond donors, six hydrogen bond acceptors, and one positive ionizable feature. These features represent non-bonding interactions between the ATX binding site and the native ligands. We applied this 17-feature pharmacophore hypothesis as a 3D probe to filter candidates from the MolPort database using the “screening” module of LigandScout. During screening, LigandScout analyzed the alignment of the drug candidates with the query features and ranked them based on the pharmacophore fit score. This pharmacophore-based screening yielded 462 hits (pharmacophore fit-score > 50), which were subsequently subjected to molecular docking-based screening against ATX, followed by LPAR1.

### 2.2. Molecular Docking-Based Virtual Screening

**Figure 4:**
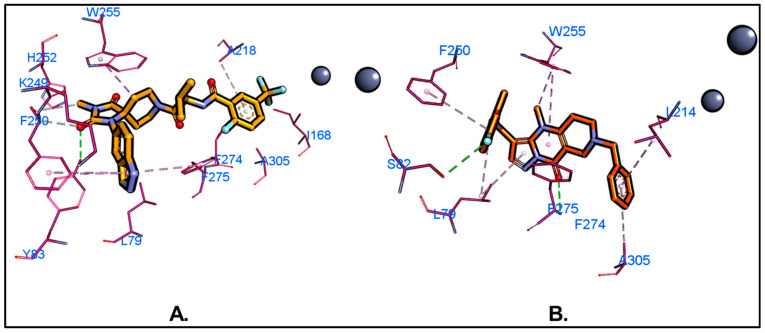
Analysis was performed on non-bonding interactions for (**A**) the natural ligand (PDB ID: 6W35) and (**B**) MolPort-137 within the ATX active site. The CPK model serves to illustrate zinc ions. In the MolPort-137 3D docked structure, carbon atoms are depicted in gold, oxygen in red, nitrogen in blue, and fluorine in cyan. Green dashed lines indicate hydrogen bonds; cyan dashed lines indicate halogen bonds, and pink dashed lines indicate other different critical interactions such as π-alkyl π-π interactions.

Molecular docking is an essential step in virtual screening, predicting the optimal orientation of a molecule within a binding site of the target protein and estimating the strength and nature of the interaction. This process involves positioning ligands into the protein’s binding pocket and evaluating their fit using scoring functions that consider various interaction forces, such as hydrogen bonds, hydrophobic interactions, and van der Waals forces. In this study, top candidates from SB pharmacophore screening (n = 462, pharmacophore fit-score ≥ 50) underwent further filtering through molecular docking-based screening. The compounds were first docked against ATX (PDB ID: 6W35) and then against LPAR1. The docking experiment was authenticated by redocking the native ligand into the protein structure of the PDB complex, and an RMSD of 0.589 Å (DockRMSD server) was obtained upon overlapping the crystal pose against the docking pose, which substantiates the reproducibility of our docking procedure (Figure S1, Supplementary Information). It was identified that the native ligand at the ATX active site was a hybrid tunnel–pocket inhibitor (Type IV). Docking with AutoDock Vina revealed several ligands with binding modes similar to the native ligand which interacted with key hydrophobic residues, including ALA218, LEU214, PHE274, PHE211, TRP261, TRP255, PHE275, and LEU79. Compounds with docking scores of −8.0 or lower, as determined by the AutoDock Vina scoring function, were classified as hits and subsequently docked against LPAR1. The top 15 compounds with the highest docking scores against both targets, ATX and LPAR1, were selected for purchase and further biological evaluation (Table S1, Supplementary Information). MolPort-137, identified as a biologically validated hit through the autotaxin inhibition experiment in vitro, formed a hydrogen bond with PHE275, a residue found between the tunnel and the hydrophobic pocket at the ATX active site. Additionally, MolPort-137 formed a halogen bond with SER82 through its fluorine atom, along with van der Waals interactions with TRP255, LEU214, and ALA305 (Figure 4). The docking pose indicated that MolPort-137 binds within the tunnel, extending slightly into the hydrophobic pocket.

### 2.3. Autotaxin Enzyme Inhibition Assay

**Figure 5:**
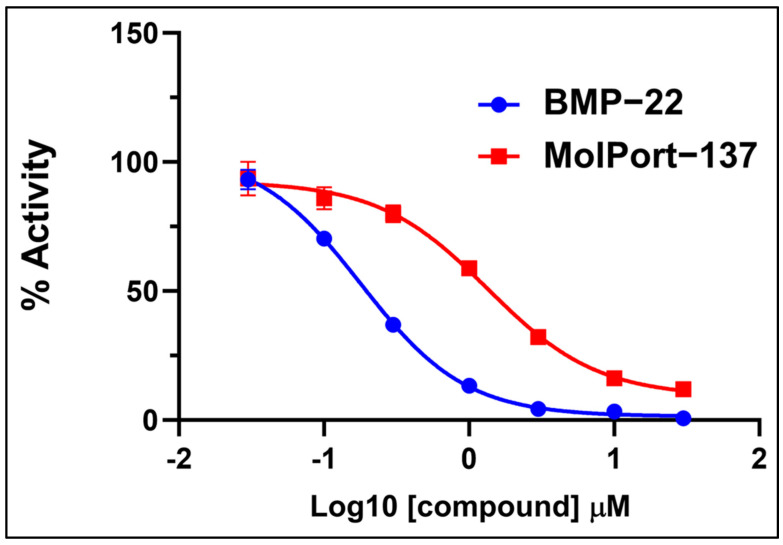
Representative dose–response curve for the FS-3-based ATX enzyme inhibition assay. The inhibitory activities of the hit compound MolPort-137 and the established ATX inhibitor BMP-22 were evaluated. The assay was carried out in biological duplicates (n = 2), with MolPort-137 achieving an average IC_50_ of 1.6 ± 0.2 μM and BMP-22 displaying an average IC_50_ of 0.2 ± 0.0 μM.

Inhibition tests were conducted to target ATX-facilitated hydrolysis of FS3 (LPC homolog) using 15 compounds at 10 μM. Compounds inhibiting more than 50% of FS-3 hydrolysis were classified as hits. Only MolPort-137 met this threshold, and it was further tested in a dose–response assay, yielding an average IC50 of 1.6 ± 0.2 μM (Figure 5). BMP-22, a known ATX inhibitor, served as a control with an IC50 of 0.2 ± 0.0 μM, consistent with reported values [35].

### 2.4. Molecular Dynamics (MD) Simulations and Binding Free Energy Calculations

**Figure 6:**
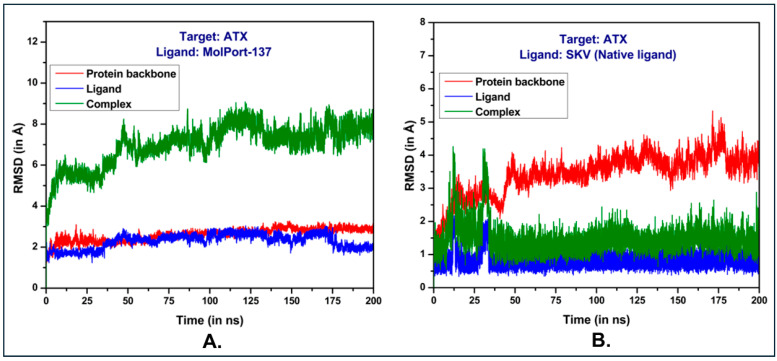
RMSD analysis of protein–ligand MD simulations for: (**A**) MolPort-137 ATX complex and (**B**) native ligand ATX complex (PDB ID: 6W35).

**Figure 7:**
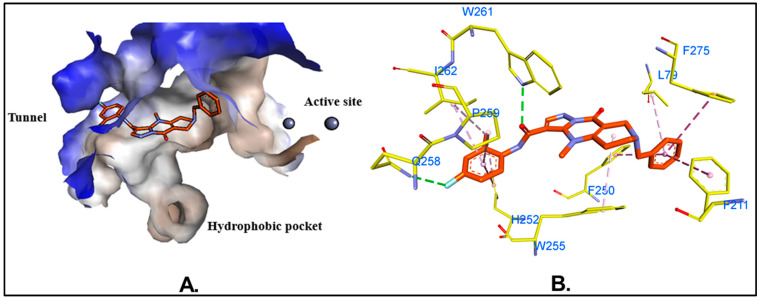
*(***A**) The average pose was obtained using the GROMOS clustering algorithm during the MD simulation. (**B**) Non-bonding interactions of MolPort-137 in its average pose with ATX. Hydrogen bonds are shown as green dashed lines, halogen bonds as cyan dashed lines, and π-π/π-alkyl interactions as pink dashed lines.

**Figure 8:**
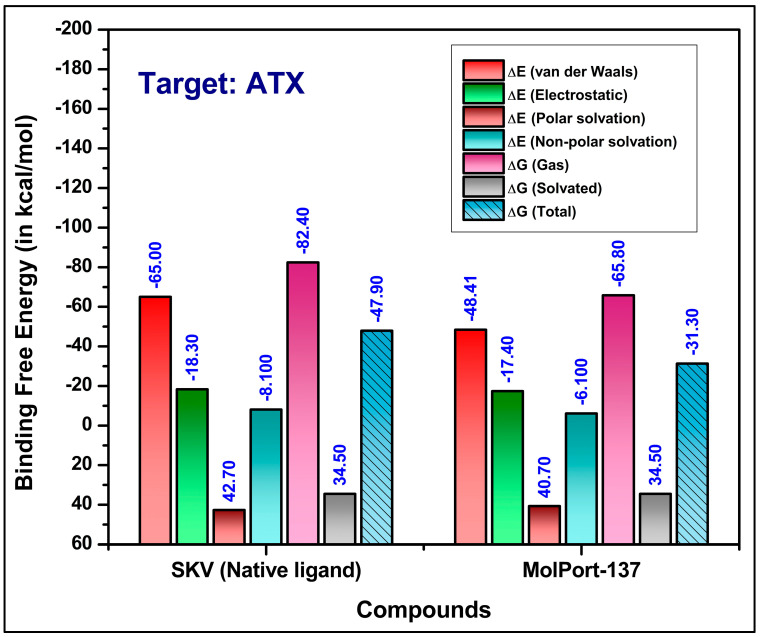
Binding free energies for the native ligand (PDB ID: 6W35) and MolPort-137 against ATX using the MM-GBSA method.

MD simulations provide a dynamic and in-depth approach to exploring molecular interactions, yielding a more precise understanding of protein–ligand interactions. This capability makes MD simulations highly valuable for identifying and refining potential drug candidates. In this study, we used MD simulations to investigate the binding dynamics and stability of MolPort-137 with autotaxin (ATX) over time. Average RMSD values of 2.3 Å for the ligand, 2.6 Å for the protein backbone, and 7.0 Å for the protein–ligand complex were attained from a 200 ns MD simulation of the MolPort-137-ATX complex (Figure 6A). In comparison, the native ligand (PDB ID: 6W35) had RMSD values of 0.9 Å, 3.4 Å, and 1.5 Å for the ligand, protein backbone, and protein–ligand complex, respectively (Figure 6B). The increased RMSD for the MolPort-137 complex suggests some movement within the active site relative to the ligand’s early docking position; however, MolPort-137 maintained stable binding within the tripartite site over the course of the simulation. Analyzing the average pose from the course of the simulation, the native ligand (PDB ID: 6W35) was shown to form hydrogen bonds with residues present in the tunnel region such as TRP255 and SER82 and a halogen bond with hydrophobic pocket residue PHE274, as well as additional π-π and van der Waals interactions with residues like LEU214, PHE250, and TRP261. In contrast, MolPort-137, in its average MD simulation pose, remained closer to the tunnel region rather than profoundly entering the hydrophobic pocket, as seen with the native ligand (Figure 7A). This positioning was reaffirmed when the MD simulation was repeated with MolPort-137’s second-best docking pose, consistently locating it within the tunnel region over the 200 ns duration (Figure S2, Supplementary Information). Furthermore, the hydrogen bond analysis reveals that MolPort-137 consistently forms one to two hydrogen bonds with ATX (Figure S3, Supplementary Information). Key interactions for MolPort-137 included hydrogen bonds with TRP261 and GLN258, as well as π-π and van der Waals contacts with residues such as TRP255, LEU79, PHE250, PHE275, PHE211, HIS252, PRO259, and ILE262 (Figure 7B). MM-GBSA binding free energy calculations showed MolPort-137 with a value of −31.30 kcal/mol, which was mainly influenced by van der Waals forces (Figure 8). In comparison, the native ligand (PDB ID: 6W35) had a stronger BFE of −50.49 kcal/mol, indicating higher affinity. The residue-level decomposition analysis of MolPort-137’s binding free energy revealed notable contributions from PHE211, PHE250, HIS252, TRP255, TRP261, ILE262, and PHE275 (Table 1). The strongest electrostatic impact came from TRP261 at −2.70 kcal/mol, owing to hydrogen bonding, while TRP255 was the main contributor to van der Waals interactions with −3.63 kcal/mol. We compared the molecular interactions of MolPort-137 with those of other ATX inhibitors and observed that MolPort-137 interacts with residues associated with the binding of Type III ATX inhibitors at the tunnel site. Specifically, amino acid residues such as PHE250, HIS252, TRP255, TRP261, and PHE275 consistently interact with Type III ATX inhibitors. This binding pattern aligns with findings reported in the comprehensive review by Salgado-Polo and Perrakis (2019) [36].

**Figure 9:**
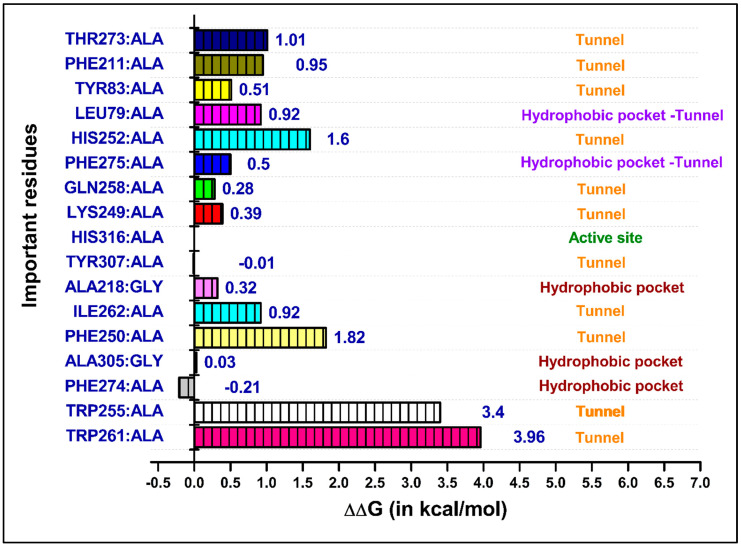
Results of the computational alanine scanning mutagenesis calculations obtained using the MM-GBSA method. ΔΔG values were determined using the following equation: ΔΔG = ΔG_mutated_ − ΔG_normal_. All the important amino acid residues, except for alanine, were mutated to alanine, and alanine residues were mutated to glycine. The amino acid residues labeled ‘Hydrophobic pocket-Tunnel’ indicate their location in the region between the hydrophobic pocket and the tunnel of the ATX binding site.

The computational alanine scanning mutagenesis provided insights into the energetic contributions of individual residues to the overall binding affinity, allowing us to identify key residues critical for the stability and binding of MolPort-137 to ATX (Figure 9). The analysis revealed that the tunnel residue TRP261 had the most substantial effect on the binding energy of MolPort-137, followed by TRP255, PHE250, HIS252, and THR273. This indicates the critical role these residues play in maintaining the stability and binding affinity of MolPort-137 with ATX. Interestingly, hydrophobic pocket residues, such as PHE274, ALA305, ALA218, and the active site residue HIS316 showed no significant impact on the binding of MolPort-137 to ATX. This observation aligns with the average pose obtained from clustering during the 200 ns MD simulation, which indicated that MolPort-137 predominantly positions itself in the tunnel region rather than deeply penetrating the hydrophobic pocket. These results confirm the crucial involvement of the tunnel residues in the binding dynamics of MolPort-137, providing valuable insights into its mechanism of action as an ATX inhibitor. The minimal impact of the hydrophobic pocket residues further supports the idea that MolPort-137 potentially acts primarily as a tunnel inhibitor.

To investigate the dual-targeting ability of MolPort-137, we conducted MD simulations against LPAR1. The average RMSDs were 3.6 Å for the protein backbone, 2.6 Å for the ligand, and 7.2 Å for the protein–ligand complex (Figure S4, Supplementary Information). MM-GBSA calculations over the last 150 ns revealed that MolPort-137 has a binding free energy of −32.50 kcal/mol, compared to −30.10 kcal/mol for the native ligand (PDB ID: 4Z35) (Table S3, Supplementary Information). Based on these findings, Molport-137 may have activity against LPAR1, and the dual-targeting ability may be further explored in the future.

Our comprehensive computational analyses reveal that MolPort-137 potentially binds at the allosteric tunnel site against ATX. The identification of key interacting residues provides insights into further optimization and enhancement of the efficacy of MolPort-137 as a therapeutic agent. The pharmacokinetic characteristics and drug-likeness of MolPort-137 were evaluated using the *SwissADME* and *pkCSM* tools [37,38]. *SwissADME* predicted drug-like attributes like lipophilicity, solubility, and absorption, while *pkCSM* assessed ADMET parameters. The results are shown in Tables S4 and S5 (Supplementary Information).

### 2.5. MolPort-137 Improves the Efficacy of PTX Treatment in Murine Breast Carcinoma Cells

**Figure 10:**
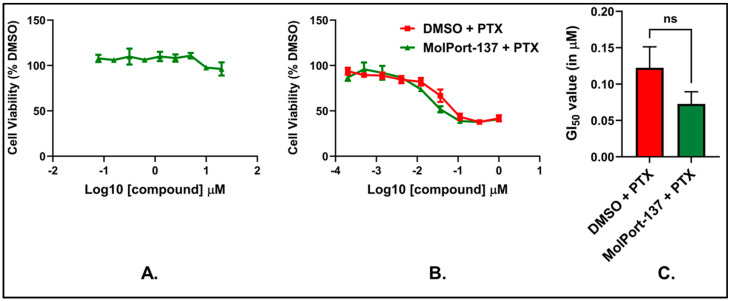
(**A**) 4T1 cells were plated with multiple serial dilutions of MolPort-137 for 72 h, and CellTiter-Glo test was used to assess cell viability. All values were normalized to DMSO-treated cells, and the GI_50_ was calculated. For MolPort-137, the GI_50_ was determined to be >20 μM, which was the highest dose tested in this assay. (**B**) 4T1 cells were plated with 3 μM MolPort-137 for 24 h, and then serial dilutions of PTX were added to each well for an additional 48 h. All values were normalized as in (**A**), and the graph shows the average cell viability obtained at each dose. (**C**) Data in (**B**) were used to calculate the GI_50_ for each condition as shown in the bar graph (n = 3 biological replicates for all assays; error bars are standard error (SE)). GraphPad Prism was employed to perform two-tailed unpaired Student’s *t*-tests. For the data in panel (**C**), the calculated *p*-value was 0.2107.

**Figure 11:**
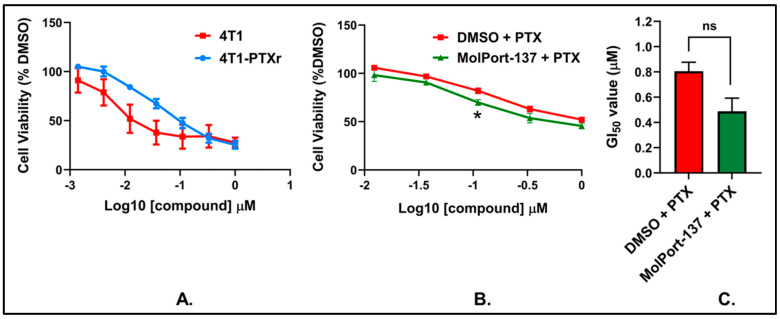
(**A**) Paclitaxel-resistant 4T1 cells were generated using dose-escalation with PTX and final 4T1-PTXr cell lines were tested for their sensitivity to PTX as compared to parental 4T1 cells. All cell lines were plated with multiple 3-fold serial dilutions of PTX for three days. Normalized values were used to determine the GI_50_ for each cell line. The 4T1 cells show a three-day GI_50_ value of 24 ± 0.9 nM, while 4T1-PTXr cells show an impaired response with a GI_50_ value of 114 ± 17 nM. (**B**) The 4T1-PTXr cells were plated with 3 μM MolPort-137 for 24 h, and then serial dilutions of PTX were added to each well for an additional 48 h. All values were normalized as in (**A**), and the graph shows the average cell viability obtained at each dose depicted. (**C**) Data in (**B**) were used to calculate the GI_50_ for each condition, as shown in the bar graph (n = 3 biological replicates for all assays; error bars are SE). GraphPad Prism was employed to perform two-tailed unpaired Student’s *t*-tests. In panel (**B**), the concentration indicated with an asterisk (*) was statistically significant (*p* < 0.05) with a *p*-value of 0.0466. In panel (**C**), the calculated *p*-value was 0.0656.

To understand the cellular activity of our experimentally validated hit, MolPort-137, we assessed the compound’s impact on cell survival against murine 4T1 mammary carcinoma cells. We chose the 4T1 breast cancer cell line for this study due to its significant relevance in evaluating autotaxin inhibitors. This cell line is known for its high tumorigenicity and metastatic potential, closely mimicking stage IV human triple-negative breast cancer, which is characterized by aggressive disease progression and distant organ metastasis. As a single agent, MolPort-137 was ineffective at impairing cell viability at all tested doses (Figure 10A), consistent with the literature that ATX inhibitors tend to be non-toxic to the cells [19,39]. Considering the function of the ATX and LPA axis in fostering chemoresistance, we next asked whether MolPort-137 could amplify the antiproliferative response induced by the chemotherapeutic agent PTX. To do so, we treated 4T1 cells for 24 h with 3 μM MolPort-137, a concentration that exhibited no cytotoxicity on its own (Figure 10A). Then, we added increasing concentrations of PTX and let cells incubate for an extra 2 days. Notably, MolPort-137 enhanced the efficacy of PTX, reducing the GI_50_ from 120 ± 27 nM for PTX alone to 69 ± 18 nM (Figure 10B,C). Because resistance to PTX treatment is common clinically, we generated 4T1 cells, called 4T1-PTXr, that show a ~5-fold increase in GI_50_ for PTX when compared to the parental 4T1 cell line (Figure 11A). Remarkably, in these cell lines, MolPort-137 also improved sensitivity to PTX treatment, reducing the GI_50_ from 788 ± 98 nM for PTX alone to 468 ± 77 nM (Figure 11B,C). This reduction in GI_50_ across all cell lines tested indicates that MolPort-137 can improve the potency of PTX, highlighting its potential as a valuable adjuvant in chemotherapeutic regimens aimed at overcoming drug resistance.

### 2.6. MolPort-137 Does Not Exhibit Cytotoxicity When Used Alone

MolPort-137 does not demonstrate cytotoxicity on its own within the tested concentration range in HaCaT keratinocytes, indicating high cellular tolerance in non-cancerous cells (Table 2). In MDA-MB-231 breast cancer cells, the compound exhibited a minimal cytotoxic effect, with a GI_50_ value of 27 ± 4 µM (Table 2). This GI_50_ value is approximately ten times higher than the concentration used in our combination treatment with PTX, suggesting that MolPort-137 is unlikely to contribute significantly to cytotoxic effects at the tested combination doses. Representative dose–response curves for each cell line are provided in Figure S5 (Supplementary Information). These findings highlight the compound’s favorable safety profile and potential suitability for further evaluation in therapeutic strategies.

### 2.7. Chemical Stability of MolPort-137

The stability of MolPort-137 was evaluated in a serum containing, cell-free media to mimic the conditions used in the synergistic assay. The compound demonstrated excellent stability, maintaining its structural integrity for 48 h (Table S6, Supplementary Information). These findings confirm that MolPort-137 is well suited for further evaluation for therapeutic purposes, ensuring reliable performance under experimental conditions.

## 3. Materials and Methods

### 3.1. Computational Details

To identify novel ATX-LPAR1 axis inhibitor hits, we employed computational tools to predict binding affinity. First, we generated a structure-based pharmacophore for the target ATX and screened the MolPort library using LigandScout 4.4 [40]. Next, the molecular docking method on the identified hits was performed with the PyRx/AutoDock Vina 0.8 software [41,42]. After validating our hit compound through in vitro enzyme inhibition studies, MD simulations were conducted using the GROMACS 2024.3 software [43] to learn more about the binding dynamics of the compound. Further evaluation of binding affinities was achieved through MM-GBSA and alanine scanning mutagenesis using the gmx_MMPBSA v1.6.4 tool [44]. The visualization of intermolecular non-bonding contacts and structures was performed with the Biovia Discovery Studio visualizer 2020 [45].

### 3.2. Generation of Structure-Based Pharmacophores

We developed a merged-feature pharmacophore for ATX using two X-ray co-crystal structures with non-covalent inhibitors (PDB IDs: 6W35 and 5MHP, with resolutions of 1.98 Å and 2.43 Å, respectively) [28,46]. Initially, the RCSB Protein Data Bank (PDB) was accessed to attain the structural coordinates of the protein–ligand complexes. The protein structures were refined by removing water and ions, repairing missing hydrogens and protein loops, and performing energy minimization with Swiss PDB-Viewer 4.1 [47]. The energy-minimized complexes were then imported into the structure-based (SB) module of LigandScout 4.4. Next, two SB pharmacophore hypotheses were generated for each protein complex. Various pharmacophore features were mapped, including hydrophobic features, hydrogen bond donors and acceptors, and other characteristics. Individual pharmacophore hypotheses were aligned using the alignment module of LigandScout 4.4. Finally, we created a merged-feature pharmacophore by combining and interpolating the overlapping features of the aligned hypotheses.

### 3.3. Virtual Screening Using Pharmacophore Models

Compounds from the MolPort library were virtually screened using the developed SB pharmacophores. LigandScout 4.4 modeling software was used for constructing the compounds’ three-dimensional structures using MMFF94 [48] energy minimization, which also assigned protonation states and produced likely stereoisomers and tautomers. The screening module of LigandScout 4.4 identified compounds with key pharmacophore features essential for binding to ATX. The compounds were initially screened using the ATX merged-feature SB pharmacophore and were finally ranked by their pharmacophore fit score, a measurement of how closely the compound structure fits with the features of the pharmacophore. Compounds with a 50% or higher pharmacophore fit score were selected as hits.

### 3.4. Molecular Docking

The compounds identified through the structure-based pharmacophore screening were put through molecular docking studies, first against ATX (PDB ID: 6W35) and then against LPAR1 (PDB ID: 4Z35) [49], using the PyRx/AutoDock Vina software. A grid box was centered on the native ligand, ensuring all hotspot residues were included, with dimensions of 10 × 10 × 10 Å. Subjecting the native ligands to docking and comparing alignment with the poses shown in the crystal structures allowed for an evaluation of the docking experiment. To ensure accuracy, the DockRMSD web server was utilized to calculate the root-mean-square deviations (RMSDs) of the docked poses from the crystal structure poses [50]. The screened hits were ranked based on their docking scores, with candidates scoring −8.0 kcal/mol or better considered as hits. These compounds were then purchased for evaluation against a human recombinant autotaxin enzyme inhibition experiment in vitro.

### 3.5. Molecular Dynamics (MD) Simulations

Molecular docking was utilized to acquire the protein–ligand complexes’ starting coordinates. The GROMACS 2024.3 software was used to carry out MD simulations, with the CHARMM36 force field [51] applied to proteins and the CHARMM general force field (CGenFF) [52] used for ligands. The complex was prepared in a dodecahedral box with TIP3P water, neutralized with Na^+^ and Cl^−^, and then minimized using 5000 steepest descents and 10,000 conjugate gradient steps. It was heated to 300 K in a 50 ps NVT ensemble, followed by 1 ns NPT equilibration at 300 K and 1 atm. MD simulations (200 ns) employed particle mesh Ewald for electrostatics and a van der Waals cutoff of 1.2 nm. Multiple replicas with different random velocities were ran, and the results were averaged. The gmx_cluster module identified the representative pose using the GROMOS algorithm with a 0.15 nm cutoff.

### 3.6. Binding Free Energy Calculations

The MM-GBSA technique was exploited to compute the binding free energy (BFE) of converged trajectories utilizing gmx_MMPBSA, considering van der Waals, electrostatics, and solvation energy components. Polar solvation was calculated with GB-OBC2 (igb = 5) [53] using dielectric constants of 1 (solute) and 78.5 (exterior dielectric constant for solvent). The non-polar solvation energy (ΔE_non-polar_) was calculated using the LCPO algorithm [54]: ΔE_non-polar_ = 0.0072 ∗ ΔSASA, with a 1.4 Å probe radius. Ions and water molecules were eliminated before the MM-GBSA analysis. The total BFEs (ΔG_Total_) of the ligands in the complex with the proteins of interest were then estimated with the comprehensive protocol described in [55].

ΔG_Total_ = ΔG_gas_ + ΔG_solv_

where

ΔG_gas_ = ΔE_van der Walls_ + ΔE_electrostatic_

and

ΔG_solv_ = ΔE_polar_ + ΔE_non-polar_

For each complex, the ligand–protein interactions were examined for each residue through a MM-GBSA decomposition analysis using the gmx_MMPBSA tool.

### 3.7. Computational Alanine Scanning Mutagenesis

To understand the role of specific residues at the protein–ligand interface in binding affinity, we executed computational alanine-scanning mutagenesis on the MolPort-137–ATX complex. MM-GBSA method was used to compute the binding free energies (BFEs) of the ligand to the protein mutants. The complex, protein, and ligand structures were derived from the same snapshot of the MD simulation trajectories. Alanine mutant structures were generated by modifying the coordinates of the normal trajectory, truncating the side chains of the mutated residues at the Cγ position, and replacing them with hydrogen atoms. The topology files were updated to reflect the alanine residue parameters for the mutated residues. In our study, each residue in the tripartite binding site of ATX was individually mutated to alanine. The MM-GBSA approach was then employed to compute the BFE differences between the mutant and normal complexes. The binding free energy difference (ΔΔG) was calculated using the following formula:

ΔΔG = ΔG_mutated_ − ΔG_normal_

In this expression, a highly positive ΔΔG value signifies a significant role of the specific residue in ligand binding.

### 3.8. Experimental Details

#### 3.8.1. Compounds/Samples

Samples of the identified hit compounds from virtual screening were procured in powder form from MolPort. Stock solutions were prepared in dimethyl sulfoxide (DMSO) at 25 mM strength. Dilutions were then prepared in assay buffer or DMSO, following their respective protocols.

#### 3.8.2. Cell-Free ATX Enzyme Inhibition Experiment

The purification of recombinant human ATX was carried out as reported earlier [56]. The potential inhibitory effect of the hits identified through molecular modeling was assessed using the FS-3 assay, which measures fluorescence when the non-natural lipid-like FRET-established substance FS-3 (Echelon Biosciences, Salt Lake City, UT, USA) is hydrolyzed by ATX. A 60 μL volume of reaction mixture containing ATX assay buffer (pH 8.0, containing 10 μM BSA, 50 mM TRIS, 140 nM NaCl, 5 mM KCl, 1 mM CaCl_2_, and 1 mM MgCl_2_) was added to each well of a black-wall 96-well plate. Recombinant ATX was then added to achieve a final concentration of 4 nM. The compounds were dissolved in DMSO and transferred to a 96-well plate in technical triplicate. Single-dose testing was performed with a final well concentration of 10 μM, while dose–response assays involved eight different concentrations of the test compound. FS-3 was then added to achieve a concluding well strength of 1 μM, and fluorescence was quantified on a CLARIOstar Plus plate reader (BMG Labtech, Ortenberg, Baden-Württemberg, Germany) for two hours, with scans every two minutes at excitation/emission wavelengths of 485/528 nm. Percent activity was calculated relative to DMSO-treated control wells. For dose–response assays, the inhibitory concentration (IC_50_) was calculated using the GraphPad Prism software 10.3.0, and the activity for each compound was reported as an average of two independent replicates ± SD.

#### 3.8.3. Cell Viability Assay

The 4T1 murine breast cancer cells were generously provided by the laboratory of Dr. Tiffany Seagroves at UTHSC. Cells were grown in RPMI-1640 media (with L-glutamine from Corning, Durham, NC, USA) complemented with 1% penicillin/streptomycin and 10% charcoal-stripped fetal bovine serum (FBS) (Gibco, ThermoFisher Scientific, Waltham, MA, USA). To generate paclitaxel-resistant 4T1 cells (4T1-PTXr), the cells were treated with escalating doses of PTX, which included 40 nM, 80 nM, 160 nM, and 250 nM treatments. Each treatment lasted one week, with fresh dosing occurring mid-week, and cells were allowed to recover for a minimum of one week between treatments. The final 4T1-PTXr cell lines were cultured in the same media as 4T1 cells with the addition of 40 nM PTX during each passage. All cells used in this study were retained at 37 °C in a humid environment with 5% carbon dioxide. To gauge the effect of MolPort-137 or PTX as a single agent on cell viability, 750 4T1 cells were plated in triplicate in a white, clear-bottomed 96-well plate with 3-fold dilutions of PTX l or 2-fold dilutions of MolPort-137. Cells treated with DMSO were included as a control. The CellTiter-Glo assay (Promega, Fitchburg, WI, USA) was used to measure cell viability following a 72 h incubation period at 37 °C in a humid 5% CO_2_ environment. A CLARIOstar plate reader (BMG Labtech, Ortenberg, Baden-Württemberg, Germany) was used to assess luminescence, and the mean values of the control wells treated with DMSO were used to normalize the data for each dose. To determine whether MolPort-137 enhances the effectiveness of PTX treatment, 4T1 or 4T1-PTXr cells were plated as described previously and treated with either 3 μM MolPort-137 or a corresponding DMSO control for 24 h. The following day, serial dilutions of PTX were applied to the previously treated cells, which were then incubated for an extra 48 h at 37 °C in a humid 5% CO_2_ environment. PTX treatment was also tested in combination with DMSO pre-treatment for comparison. As described in the single agent assay, the CellTiter-Glo assay was used to evaluate cell viability in the synergistic assay. GraphPad Prism (GraphPad Software, version 10.0.2 (232), San Diego, CA, USA) was used to analyze and visualize the data. A nonlinear regression analysis, based on the inhibitor vs. normalized response dose–response inhibition model, was performed on all viability data to calculate the GI_50_ concentrations for MolPort-137 both alone and in combination with PTX.

The cytotoxic effects of MolPort-137 were further evaluated using the CellTiter-Glo assay in two cell lines: MDA-MB-231 breast cancer cells and HaCaT keratinocytes. Both cell lines were grown in DMEM medium (Corning, Durham, NC, USA) containing L-glutamine, accompanied by 10% fetal bovine serum (FBS) (Gibco, Thermo Fisher Scientific, Waltham, MA, USA) and 1% penicillin-streptomycin. For the assay, 2000 MDA-MB-231 cells and 5000 HaCaT cells were seeded in triplicate into individual wells of a white, clear-bottomed 96-well plate. MolPort-137 was tested at concentrations ranging up to 100 µM. DMSO-treated cells served as the control. Following 72 h of incubation at 37 °C in a humid 5% CO_2_ environment, cell viability was determined using the CellTiter-Glo assay (Promega, Fitchburg, WI, USA). Luminescence was measured using a CLARIOstar plate reader (BMG Labtech, Ortenberg, Germany). The luminescence values for every treatment dose were standardized against the average values of the DMSO-treated control wells to assess cell viability.

#### 3.8.4. In Vitro Chemical Stability Study

The in vitro stability of MolPort-137 was assessed using an assay medium comprising RPMI-1640 supplemented with fetal bovine serum (FBS) and penicillin/streptomycin, reflecting the conditions used in combination treatment studies with 4T1 cells. The analysis was conducted at BioDuro-Sundia, Irvine, CA, USA. For each time point, 1 µL of the working solution of MolPort-137 was added to 99 µL of cell-free culture medium and mixed thoroughly by pipetting. The samples were sealed with a protective film under a biosafety cabinet and incubated at 37 °C in a 5% CO_2_ atmosphere. At the designated intervals (0, 8, 24, and 48 h), 400 µL of quenching solution was added to 100 µL of the reaction mixture, followed by vortexing for 1 min. The samples were then centrifuged at 4000 rpm for 15 min at 4 °C. The resulting supernatant (100 µL) was mixed with an equal volume of distilled water and analyzed via LC-MS/MS to evaluate the compound’s stability under the specified conditions.

## 4. Conclusions

Studies indicate that resistance to paclitaxel occurs in approximately 30–70% of patients with advanced breast cancer, leading to worse prognoses and reduced overall survival. Efforts to inhibit ATX are underway in both preclinical and clinical studies to disrupt this key signaling network and enhance treatment outcomes for cancer patients. This study demonstrates the effectiveness of a comprehensive virtual screening approach, integrating structure-based pharmacophore modeling and molecular docking, to identify novel ATX inhibitors. Through this approach, we discovered MolPort-137, which exhibited an IC_50_ of 1.6 ± 0.2 μM in an in vitro autotaxin enzyme inhibition assay. Furthermore, pre-treatment with MolPort-137 led to enhancements in paclitaxel’s potency against 4T1 murine breast carcinoma cells in a synergistic assay. Notably, it also resensitized taxol-resistant 4T1 cells to paclitaxel treatment, highlighting its potential to enhance the effectiveness of combination therapies aimed at overcoming cancer treatment resistance. Our in-depth in silico testing, including MD simulations, MM-GBSA calculations, and computational alanine scanning, revealed that MolPort-137 binds within the tunnel region of ATX. These analyses provided valuable insights into the pharmacophores of MolPort-137, and they will guide future structural modifications to enhance its inhibitory potential. In summary, our integrative computational and experimental approach identified a novel ATX inhibitor and underscored the potential of MolPort-137 in improving breast cancer therapy outcomes.

## Figures and Tables

**Table 1:** MM-GBSA-derived contributions of each binding site amino acid residue to the total binding free energy for MolPort-137 against ATX. The energy values are provided in kcal/mol.

Residues	Van Der Waals		Electrostatic		Polar Solv.		Non-Polar Solv.		TOTAL	
	Avg.	Std. Err. of Mean	Avg.	Std. Err. of Mean	Avg.	Std. Err. of Mean	Avg.	Std. Err. of Mean	Avg.	Std. Err. of Mean
LEU:79	−1.01	0.00	−0.19	0.00	0.42	0.00	−0.88	0.00	−1.67	0.01
SER:82	−0.27	0.00	−0.15	0.00	0.13	0.00	−0.24	0.00	−0.54	0.00
TYR:83	−0.31	0.00	−0.42	0.01	0.29	0.00	−0.20	0.00	−0.65	0.01
PHE:211	−1.36	0.00	0.01	0.00	0.30	0.00	−1.09	0.00	−2.14	0.00
LEU:214	−0.33	0.00	0.18	0.00	−0.15	0.00	−0.23	0.00	−0.54	0.00
TYR:215	−1.14	0.00	0.20	0.00	−0.08	0.00	−0.78	0.00	−1.80	0.00
PHE:242	−0.20	0.00	0.11	0.00	−0.06	0.00	−0.06	0.00	−0.21	0.00
LEU:244	−0.94	0.00	−0.17	0.00	0.33	0.00	−0.83	0.00	−1.62	0.00
LYS:249	−0.88	0.00	−0.06	0.01	−0.07	0.01	−0.64	0.00	−1.65	0.01
PHE:250	−2.05	0.01	−0.16	0.00	0.36	0.00	−1.47	0.00	−3.32	0.01
ASN:251	−0.23	0.00	−0.10	0.00	0.10	0.00	−0.04	0.00	−0.26	0.00
HIS:252	−1.63	0.01	−0.89	0.01	0.41	0.01	−1.47	0.01	−3.57	0.01
TRP:255	−3.63	0.01	−0.70	0.00	0.50	0.00	−2.55	0.00	−6.37	0.01
PRO:259	−1.02	0.00	−0.18	0.00	0.40	0.00	−0.61	0.00	−1.41	0.01
TRP:261	−1.70	0.01	−2.70	0.01	0.88	0.00	−1.19	0.00	−4.71	0.01
ILE:262	−1.19	0.01	−0.32	0.00	0.47	0.00	−0.98	0.01	−2.02	0.01
PHE:274	−0.58	0.00	−0.25	0.01	0.09	0.00	−0.25	0.00	−0.99	0.01
PHE:275	−1.51	0.00	0.17	0.00	−0.03	0.00	−1.16	0.00	−2.53	0.01

**Table 2:** To evaluate cytotoxicity, the relevant cell lines were plated in triplicate and subjected to three days of treatment with nine serial dilutions of either PTX or MolPort-137. Cell viability was measured using the CellTiter-Glo assay. The GI_50_ ± SE values for each agent are summarized below. Data are indicative of the average of three individual biological replicates for both MolPort-137 and PTX.

MDA-MB-231 Cells		HaCaT Keratinocytes	
Compounds	GI_50_	Compounds	GI_50_
MolPort-137	27 ± 2 µM	MolPort-137	>100 µM
PTX	02 ± 0 nM	PTX	02 ± 0 nM

## Data Availability

The data supporting this article have been included in the main text or as part of the Supplementary Information. The computational chemistry files, including molecular docking output files, molecular dynamics simulation trajectories and output files, SAPT files, and conceptual DFT files, can be made available on request.

## Supplementary Materials

The following supporting information can be downloaded at the following website: https://www.mdpi.com/article/10.3390/ijms26020597/s1.

## Abbreviations

ATX, autotaxin; LPAR, lysophosphatidic acid receptor; LPA, lysophosphatidic acid; ENPP2, ectonucleotide pyrophosphatase/phosphodiesterase 2; PTX, paclitaxel; NRF2, nuclear factor erythroid 2-related factor 2; ERK, extracellular signal-regulated kinase; PI3K, phosphoinositide 3-kinase; HDAC, histone deacetylase; LPC, lysophosphatidylcholine; ff99SB, Force Field 1999 Side-Chain and Backbone; MD, molecular dynamics; MM-GBSA, molecular mechanics/generalized Born surface area; GROMACS, GROningen MAchine for Chemical Simulations; PDB, protein data bank; SB, structure-based; MMFF94, Merck Molecular Force Field 1994; RMSD, root–mean–square deviation; CHARMM36, Chemistry at Harvard Macromolecular Mechanics (version 36); GROMOS96, GROningen MOlecular Simulation 1996; TIP3P, Three Point Charge Model (TIP3P); NVT, number of particles (N)–volume (V)–temperature (T); NPT, number of particles–pressure–temperature; SASA, solvent-accessible surface area; LCPO, Linear Combination of Pairwise Overlaps; hATX, human recombinant ATX; FRET, Fluorescence resonance energy transfer; FBS, fetal bovine serum; SD, standard deviation; SE, standard error.
