# Supplementary material for: Structure-Based Discovery of MolPort-137: A Novel Autotaxin Inhibitor That Improves Paclitaxel Efficacy
Source: Int J Mol Sci. 2025 Jan 12;26(2):597. doi: 10.3390/ijms26020597 (PMC11765394; doi:10.3390/ijms26020597)
Supplement: Supplementary file 1 [file ijms-26-00597-s001.zip › ijms-3410416-supplementary.pdf]

Structure-Based Discovery of MolPort-137: A Novel Autotaxin Inhibitor That Improves Paclitaxel Efficacy

Prateek Rai [1,2,†], Christopher J. Clark [1,2,†], Vandana Kardam [3], Carl B. Womack [4], Joshua Thammathong [2], Derek D. Norman [5], Gábor J. Tigyi [5], Kevin Bicker [1,2], April M. Weissmiller [1,4], Kshatresh Dutta Dubey [3,*] and Souvik Banerjee [1,2,*]

[1] Molecular Biosciences, Middle Tennessee State University, Murfreesboro, TN 37132, USA
[2] Department of Chemistry, Middle Tennessee State University, Murfreesboro, TN 37132, USA
[3] Department of Chemistry, Shiv Nadar Institution of Eminence, Delhi 201314, India
[4] Department of Biology, Middle Tennessee State University, Murfreesboro, TN 37132, USA
[5] Department of Physiology, University of Tennessee Health Science Center, Memphis, TN 37132, USA;
* Correspondence: kshatresh.dubey@snu.edu.in (K.D.D.); souvik.banerjee@mtsu.edu (S.B.)
† These authors contributed equally to this work.

Figure S1: Superimposition of the crystal pose (yellow stick) and the docking pose (pink stick) demonstrates the accuracy of the docking procedure. Zinc ions are depicted using the CPK model. A control study was conducted to validate this process, confirming that the docked pose of the native ligand aligns well with its crystal structure. The root-mean-square deviation (RMSD) between the crystal and docking poses was calculated to be 0.589 Å using the DockRMSD webserver, as detailed in the main text.

Figure S2: The average pose was obtained from the GROMOS clustering analysis from the MD simulation, starting from the second pose generated from molecular docking of MolPort-137.

Figure S3: Number of hydrogen bonds determined between the protein and the ligand for MolPort-137 throughout the 200 ns production run.

Figure S4: RMSDs obtained from the MD simulations for: **A.** native ligand (PDB ID: 4Z35) and **B.** MolPort-137 against LPAR1.

Figure S5: Representative dose-response curves from the cell viability assay using CellTiter-Glo: (A) MDA-MB-231 cancer cells and (B) HaCaT keratinocytes.

Table S1: SMILES notation, AutoDock Vina binding energies (in kcal/mol) against PDB ID: 6W35 (ATX) and PDB ID: 4Z35 (LPAR1).

Table S2: % inhibition of top 15 compounds tested at 10 µM concentration.

Table S3: Results of MM-GBSA calculations for the native ligand (PDB ID: 4Z35) and MolPort-137 against LPAR1. The energy values are given in kcal/mol.

Table S4: Evaluation of pharmacokinetics, drug-likeness, and medicinal chemistry friendliness predictions of MolPort-137 through the SwissADME webserver.

Table S5: Prediction of ADMET properties of MolPort-137 through the pkCSM webserver based on graph-based signatures.

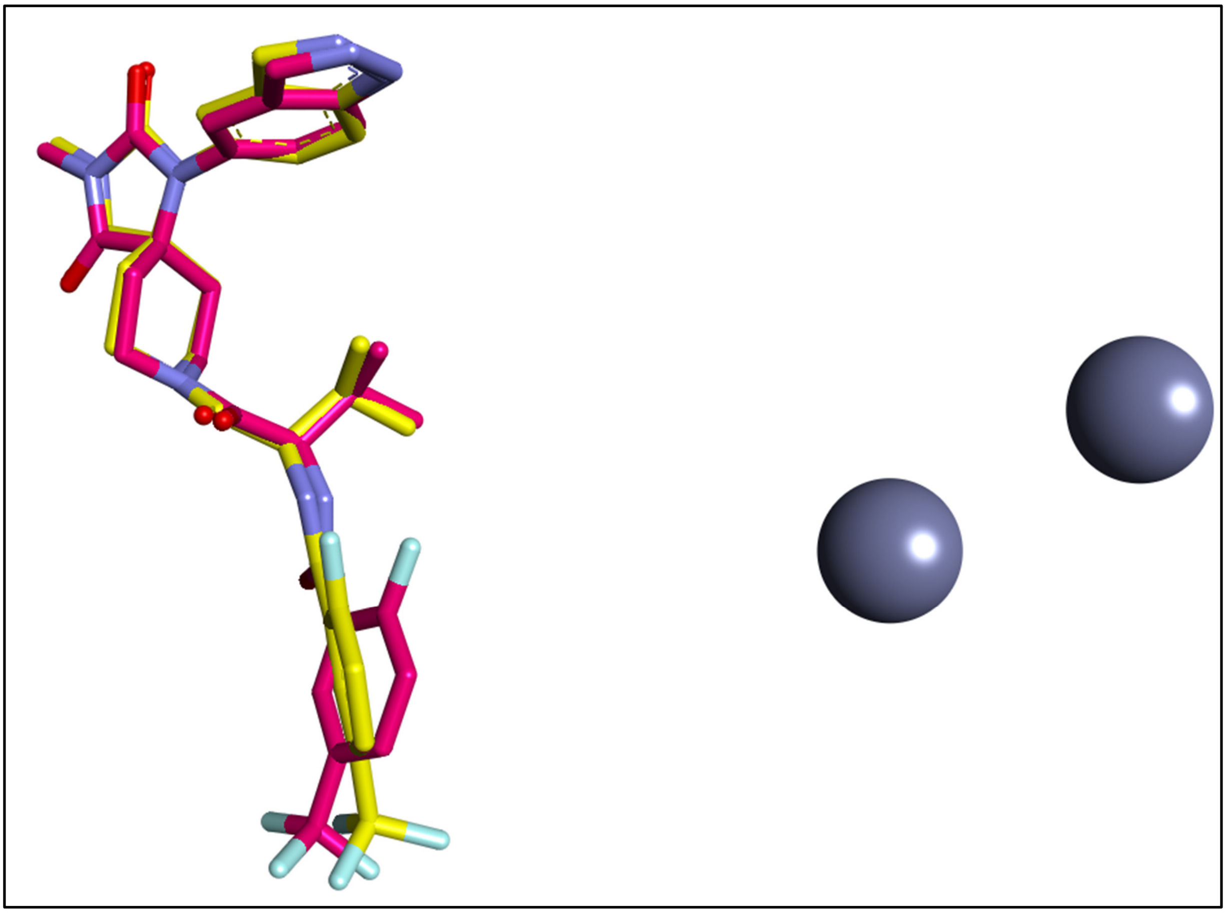

Figure S2: Superimposition of the crystal pose (yellow stick) and the docking pose (pink stick) demonstrates the accuracy of the docking procedure. Zinc ions are depicted using the CPK model. A control study was conducted to validate this process, confirming that the docked pose of the native ligand aligns well with its crystal structure. The root-mean-square deviation (RMSD) between the crystal and docking poses was calculated to be 0.589 Å using the DockRMSD webserver, as detailed in the main text.

Table S1: SMILES notation, AutoDock Vina binding energies (in kcal/mol) against PDB ID: 6W35 (ATX) and PDB ID: 4Z35 (LPAR1).

Compound name	SMILES	ATX docking score	LPAR1 docking score
MolPort-674	CCC1=CC(=CC=C1)NC(=O)CN2N=C3N(C2=O)C4=C(C=CC=C4)N=C3OC5=C(C)C=CC=C5	-12.7	-11.7
MolPort-311	CC1=C(Br)C=CC(=C1)NC(=O)CN2NC3=C(OC4=C(C)C=CC=C4)N=C5C=CC=CC5=[NH]3C2=O	-12.6	-11.6
MolPort-161	CC1=CC(=CC=C1)NC(=O)CN2N=C3N(C2=O)C4=C(C=CC=C4)N=C3OC5=C(C)C=CC=C5	-12.6	-11.3
MolPort-122	CC1=C(C[N]2N=NC(=C2C)C3=NC(=NO3)C4=CC(=C(C)C=C4)C)N=C(O1)C5=CC=CC=C5	-12.6	-10.9
MolPort-904	CC1=CC(=CC(=C1)C)[N]2C(=NC3=C2N=CC=C3)CN4CCN(CC4)C(=O)NC5=C(F)C=CC=C5	-12.7	-10.9
MolPort-839	COC1=C(OCC2=CC(=CC=C2)C)C(=CC(=C1)C=C3N=C(OC3=O)C4=CC=C(F)C=C4)I	-11.9	-10.8
MolPort-616	CC1CCCN(C1)C2=NC(=O)N(CC(=O)NC3=CC(=CC(=C3)C)C)C4=CC=CC=C24	-11.6	-10.8
MolPort-072	COC1=CC=C(NC(=O)CSC2=NC(=O)C3=C(N=C(C)C=C3C)N2C4=C(C)C(=CC=C4)C)C=C1	-11.7	-10.6
MolPort-185	COC1=C(C=CC=C1)C2=NC(=C(C)O2)C[N]3N=NC(=C3C)C4=NC(=NO4)C5=CC=C(C)C=C5	-11.6	-10.6
MolPort-914	CC1=CC(=CC=C1)CNC(=O)C2=C(O)C3=C(N=CC=C3)N(C2=O)C4=CC(=CC=C4)F	-11.9	-10.6
MolPort-495	CC1=C(C[N]2N=NC(=C2C)C(=O)NC3=C(C)C=CC=C3)N=C(O1)C4=CC(=CC=C4)Cl	-11.8	-10.4
MolPort-240	CC1=C(C)C=C(NC(=O)CN2N=C3N(C2=O)C4=C(C=CC=C4)N=C3OC5=C(C)C(=CC=C5)C)C=C1	-13.1	-10.4
MolPort-137	CN1C2=C(CN(CC2)CC3=CC=CC=C3)C(=O)[N]4N=CC(=C14)C(=O)NC5=C(C)C=C(F)C=C5	-11.6	-10.4
MolPort-323	CC1=C2C(=N)N(C3=CC(=C(Cl)C=C3)C)C(=NC2=N[NH]1)SCC(=O)NC4=CC(=C(F)C=C4)F	-11.7	-10.3
MolPort-144	CC1=CC=CC(=C1)N2C(=N)C3=C([NH]N=C3C)N=C2SCC(=O)NC4=CC(=C(C)C=C4)C	-12.4	-10.2

Table S2: % inhibition of top 15 compounds tested at 10 μM concentration.

Compound name	% inhibition in ATX activity assay at 10 μM
MolPort-674	7.10
MolPort-311	5.23
MolPort-161	5.16
MolPort-122	15.48
MolPort-904	29.67
MolPort-839	10.81
MolPort-616	26.45
MolPort-072	42.00
MolPort-185	33.70
MolPort-914	7.61
MolPort-495	27.24
MolPort-240	2.06
**MolPort-137**	**75.89**
MolPort-323	5.00
MolPort-144	-13.50

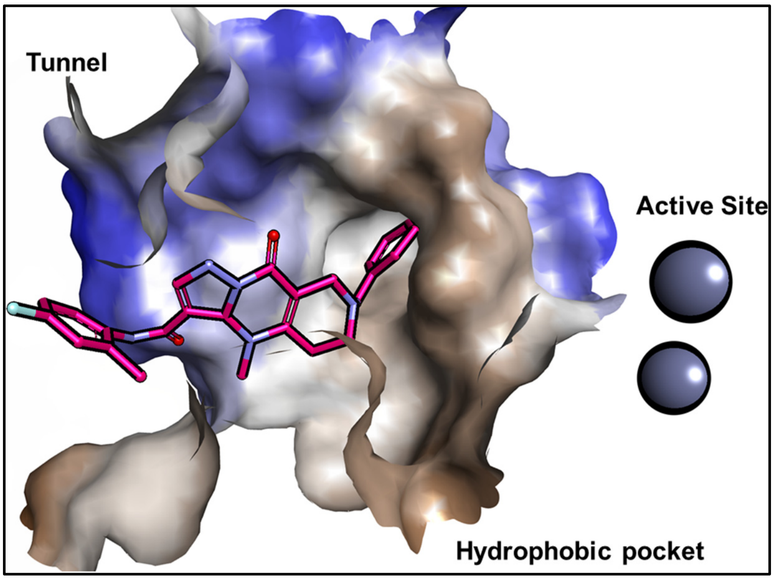

Figure S2: The average pose was obtained from the GROMOS clustering analysis from the MD simulation, starting from the second pose generated from molecular docking of MolPort-137.

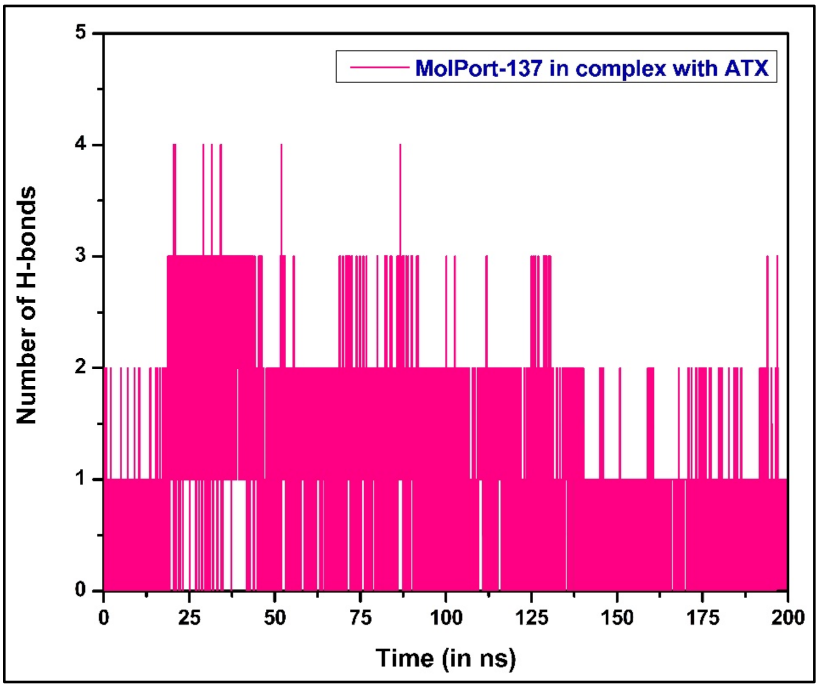

Figure S3: Number of hydrogen bonds determined between the protein and the ligand for MolPort-137 throughout the 200 ns production run.

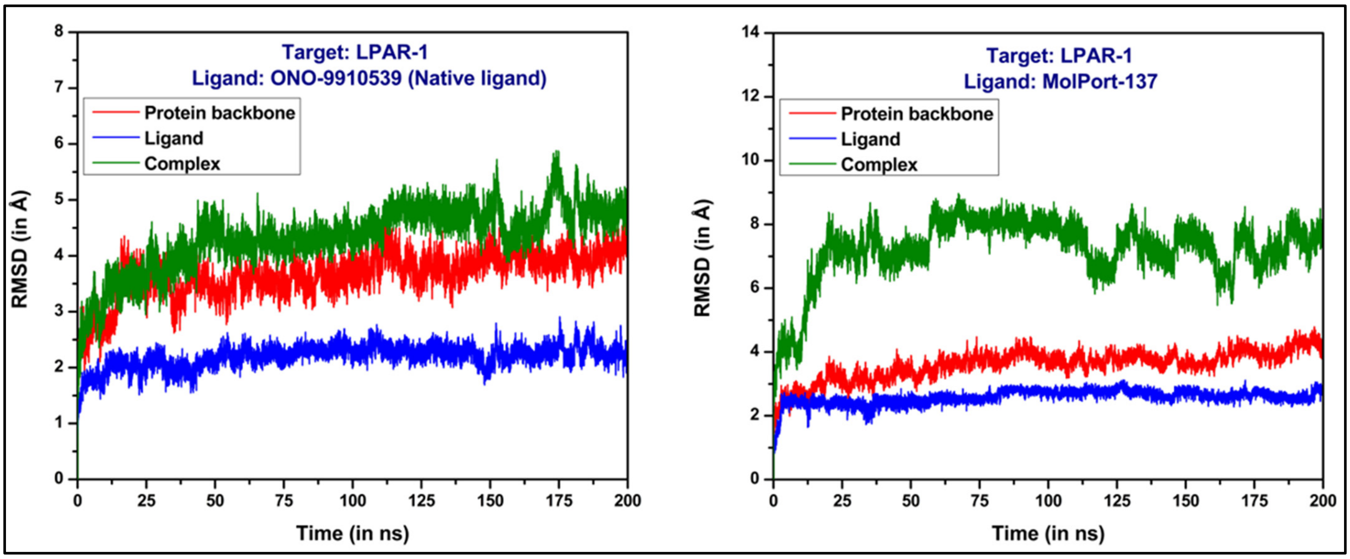

Figure S4: RMSDs obtained from the MD simulations for: **A.** native ligand (PDB ID: 4Z35) and **B.** MolPort-137 against LPAR1.

Table S3: Results of MM-GBSA calculations for the native ligand (PDB ID: 4Z35) and MolPort-137 against LPAR1. The energy values are given in kcal/mol.

Compounds	van der Waals	Electrostatics	Polar Solv.	Non-Polar Solv.	TOTAL
ONO-9910539 (native ligand) vs. LPAR1	-52.90	-17.70	48.30	-7.8	-30.10
MolPort-137 vs. LPAR1	-49.90	-1.40	24.60	-5.8	-32.50

Table S4: Evaluation of pharmacokinetics, drug-likeness, and medicinal chemistry friendliness predictions of MolPort-137 through the *SwissADME* webserver.

Molecule Name	MolPort-137
**Physicochemical properties**	
Canonical SMILES	Fc1ccc(c(c1)C)NC(=O)c1cnn2c1n(C)c1CCN(Cc1c2=O)Cc1ccccc1
Formula	C25H24FN5O2
MW	445.49
#Heavy atoms	33
#Aromatic heavy atoms	21

Fraction Csp3	0.21
#Rotatable bonds	5
#H-bond acceptors	5
#H-bond donors	1
MR	128.58
TPSA	71.64
**Lipophilicity**	
iLOGP	3.4
XLOGP3	3.13
WLOGP	2.84
MLOGP	3.32
Silicos-IT Log P	3.28
Consensus Log P	3.19
**Water solubility**	
ESOL Log S	-4.71
ESOL Solubility (mg/ml)	8.59e-03
ESOL Solubility (mol/l)	1.93e-05
ESOL Class	Moderately soluble
Ali Log S	-4.3
Ali Solubility (mg/ml)	2.21e-02
Ali Solubility (mol/l)	4.97e-05
Ali Class	Moderately soluble
Silicos-IT LogSw	-7.33
Silicos-IT Solubility (mg/ml)	2.06e-05
Silicos-IT Solubility (mol/l)	4.63e-08
Silicos-IT class	Poorly soluble
**Pharmacokinetics**	

GI absorption	High
BBB permeant	Yes
Pgp substrate	Yes
CYP1A2 inhibitor	No
CYP2C19 inhibitor	Yes
CYP2C9 inhibitor	Yes
CYP2D6 inhibitor	Yes
CYP3A4 inhibitor	Yes
log Kp (cm/s)	-6.8
**Druglikeness**	
Lipinski #violations	0
Ghose #violations	0
Veber #violations	0
Egan #violations	0
Muegge #violations	0
Bioavailability Score	0.55
**Medicinal Chemistry**	
PAINS #alerts	0
Brenk #alerts	0
Leadlikeness #violations	1
Synthetic Accessibility	3.49

Table S5: Prediction of ADMET properties of MolPort-137 through the *pkCSM* webserver based on graph-based signatures.

Property	Model Name	Predicted value	Unit
Absorption	Water solubility	-5.644	Numeric (log mol/L)
Absorption	Caco2 permeability	0.991	Numeric (log Papp in 10^{-6} cm/s)
Absorption	Intestinal absorption (human)	98.6	Numeric (% Absorbed)
Absorption	Skin Permeability	-3.044	Numeric (log Kp)
Absorption	P-glycoprotein substrate	Yes	Categorical (Yes/No)

**Absorption**	P-glycoprotein I inhibitor	Yes	Categorical (Yes/No)
**Absorption**	P-glycoprotein II inhibitor	No	Categorical (Yes/No)
**Distribution**	VDss (human)	0.405	Numeric (log L/kg)
**Distribution**	Fraction unbound (human)	0.319	Numeric (Fu)
**Distribution**	BBB permeability	-0.475	Numeric (log BB)
**Distribution**	CNS permeability	-2.972	Numeric (log PS)
**Metabolism**	CYP2D6 substrate	No	Categorical (Yes/No)
**Metabolism**	CYP3A4 substrate	Yes	Categorical (Yes/No)
**Metabolism**	CYP1A2 inhibitor	No	Categorical (Yes/No)
**Metabolism**	CYP2C19 inhibitor	Yes	Categorical (Yes/No)
**Metabolism**	CYP2C9 inhibitor	No	Categorical (Yes/No)
**Metabolism**	CYP2D6 inhibitor	No	Categorical (Yes/No)
**Metabolism**	CYP3A4 inhibitor	No	Categorical (Yes/No)
**Excretion**	Total Clearance	0.833	Numeric (log ml/min/kg)
**Excretion**	Renal OCT2 substrate	No	Categorical (Yes/No)
**Toxicity**	AMES toxicity	No	Categorical (Yes/No)
**Toxicity**	Max. tolerated dose (human)	-1.005	Numeric (log mg/kg/day)
**Toxicity**	hERG I inhibitor	No	Categorical (Yes/No)
**Toxicity**	hERG II inhibitor	No	Categorical (Yes/No)
**Toxicity**	Oral Rat Acute Toxicity (LD50)	2.92	Numeric (mol/kg)
**Toxicity**	Oral Rat Chronic Toxicity (LOAEL)	1.177	Numeric (log mg/kg_bw/day)
**Toxicity**	Hepatotoxicity	Yes	Categorical (Yes/No)
**Toxicity**	Skin Sensitisation	No	Categorical (Yes/No)
**Toxicity**	T.Pyriformis toxicity	0.429	Numeric (log ug/L)
**Toxicity**	Minnow toxicity	1.793	Numeric (log mM)

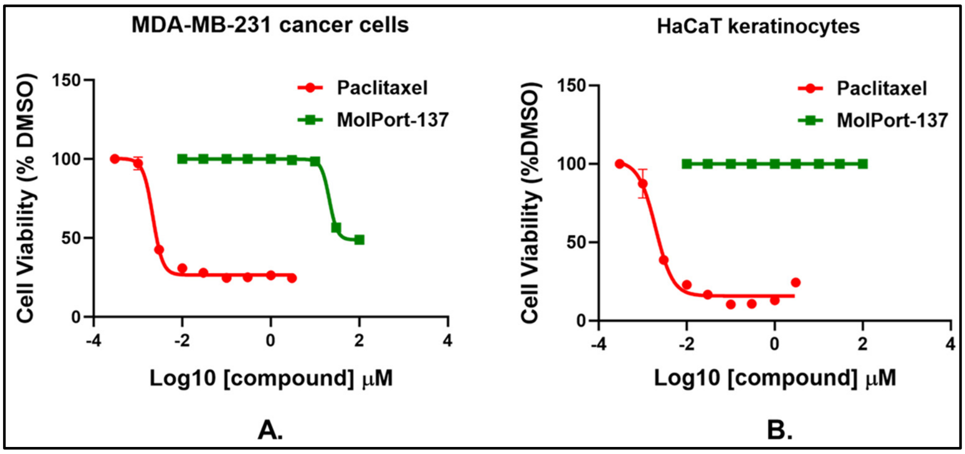

Figure S5: Representative dose-response curves from the cell viability assay using CellTiter-Glo: (A) MDA-MB-231 cancer cells and (B) HaCaT keratinocytes.

Table S6: LC-MS/MS analysis results were obtained at different time intervals to assess the chemical stability of the compound.

Time (h)	Peak Area Ratio-1	Peak Area Ratio-2	Peak Area Ratio-3	Mean	% Remaining
0	2.42E-01	2.34E-01	2.26E-01	2.34E-01	100.00
8	2.54E-01	2.26E-01	2.31E-01	2.37E-01	101.28
24	2.61E-01	2.48E-01	2.29E-01	2.46E-01	105.13
48	2.36E-01	2.46E-01	2.30E-01	2.37E-01	101.42
